# Chlorido[1*H*-1,2,4-triazole-5(4*H*)-thione-κ*S*]bis­(triphenyl­phosphane-κ*P*)copper(I) acetronitrile monosolvate

**DOI:** 10.1107/S1600536812049537

**Published:** 2012-12-08

**Authors:** Kofsoh Wani, Chaveng Pakawatchai, Saowanit Saithong

**Affiliations:** a Department of Chemistry and Center of Excellence for Innovation in Chemistry, Faculty of Science, Prince of Songkla University, Hat Yai, Songkhla 90112, Thailand

## Abstract

In the title solvate, [CuCl(C_2_H_3_N_3_S)(C_18_H_15_P)_2_]·CH_3_CN, the Cu^I^ ion is bonded to two triphenyl­phosphane ligands, one 1*H*-1,2,4-triazole-5(4*H*)-thione ligand *via* its S atom and one chloride ion in a distorted CuP_2_SCl tetra­hedron. An intra­molecular N—H⋯Cl hydrogen bond, which closes an *S*(6) ring, helps to establish the conformation of the complex. In the crystal, N—H⋯Cl hydrogen bonds and C—H⋯π inter­actions link the components, generating (110) layers.

## Related literature

For the properties of mixed-ligand copper(I) complexes, see: Oshio *et al.* (1996 ▶); Henary *et al.* (1997 ▶); Vitale & Ford (2001 ▶); Zhang & Chen (2003 ▶). For structurally related mixed-ligand complexes of triphenyl­phosphane and thione ligands, see: Skoulika *et al.* (1991 ▶); Aslanidis *et al.* (1998 ▶); Chen *et al.* (2001 ▶); Li *et al.* (2004 ▶); Lobana *et al.* (2008 ▶); La-o *et al.* (2009 ▶). For complexes of 1,2,4-triazole-2-thione and its derivatives, see: Sen *et al.* (1996 ▶); Zhang *et al.* (2008 ▶).
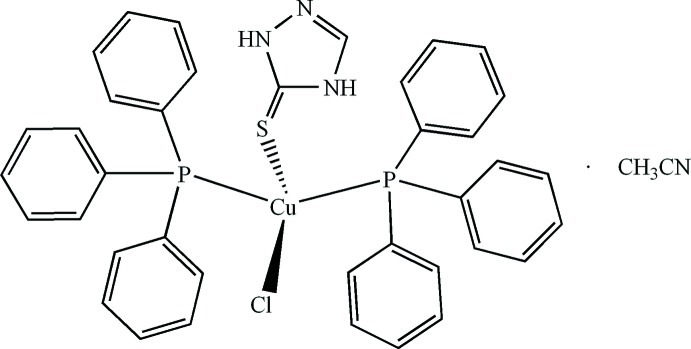


## Experimental

### 

#### Crystal data

[CuCl(C_2_H_3_N_3_S)(C_18_H_15_P)_2_]·C_2_H_3_N
*M* *_r_* = 765.72
Orthorhombic, 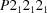

*a* = 10.2348 (4) Å
*b* = 16.4046 (7) Å
*c* = 22.3632 (9) Å
*V* = 3754.7 (3) Å^3^
*Z* = 4
Mo *K*α radiation
μ = 0.83 mm^−1^
*T* = 293 K
0.27 × 0.18 × 0.09 mm

#### Data collection

Bruker APEX CCD diffractometer
Absorption correction: multi-scan (*SADABS*; Bruker, 2003 ▶) *T*_min_ = 0.840, *T*_max_ = 0.928
30968 measured reflections
6602 independent reflections
6040 reflections with *I* > 2σ(*I*)
*R*_int_ = 0.044

#### Refinement

*R*[*F* ^2^ > 2σ(*F* ^2^)] = 0.038
*wR*(*F* ^2^) = 0.086
*S* = 1.11
6602 reflections
449 parameters
2 restraints
H atoms treated by a mixture of independent and constrained refinement
Δρ_max_ = 0.52 e Å^−3^
Δρ_min_ = −0.17 e Å^−3^
Absolute structure: Flack (1983 ▶), 2890 Friedel pairs
Flack parameter: −0.001 (11)

### 

Data collection: *SMART* (Bruker, 2003 ▶); cell refinement: *SAINT* (Bruker, 2003 ▶); data reduction: *SAINT* (Bruker, 2003 ▶); program(s) used to solve structure: *SHELXS97* (Sheldrick, 2008 ▶); program(s) used to refine structure: *SHELXL97* (Sheldrick, 2008 ▶); molecular graphics: *Mercury* (Macrae *et al.*, 2008 ▶); software used to prepare material for publication: *SHELXTL* (Sheldrick, 2008 ▶), *PLATON* (Spek, 2009 ▶) and *publCIF* (Westrip, 2010 ▶).

## Supplementary Material

Crystal structure: contains datablock(s) I, global. DOI: 10.1107/S1600536812049537/hb7008sup1.cif

Structure factors: contains datablock(s) I. DOI: 10.1107/S1600536812049537/hb7008Isup2.hkl

Additional supplementary materials:  crystallographic information; 3D view; checkCIF report

## Figures and Tables

**Table 1:** Selected bond lengths (Å)

Cu1—P1	2.2802 (9)
Cu1—P2	2.2824 (9)
Cu1—S1	2.3582 (9)
Cu1—Cl1	2.4035 (9)

**Table 2:** Hydrogen-bond geometry (Å, °) *Cg*7 is the centroid of the C31–C36 ring.

*D*—H⋯*A*	*D*—H	H⋯*A*	*D*⋯*A*	*D*—H⋯*A*
N3—H3*A*⋯Cl1	0.84 (2)	2.41 (3)	3.183 (3)	155 (5)
N1—H1*A*⋯Cl1^i^	0.84 (2)	2.34 (2)	3.154 (3)	163 (5)
C15—H15⋯*Cg*7^ii^	0.93	2.88	3.749 (4)	155

Symmetry codes: (i) 
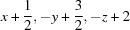
; (ii) 
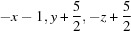
.

**Fig. 1.**
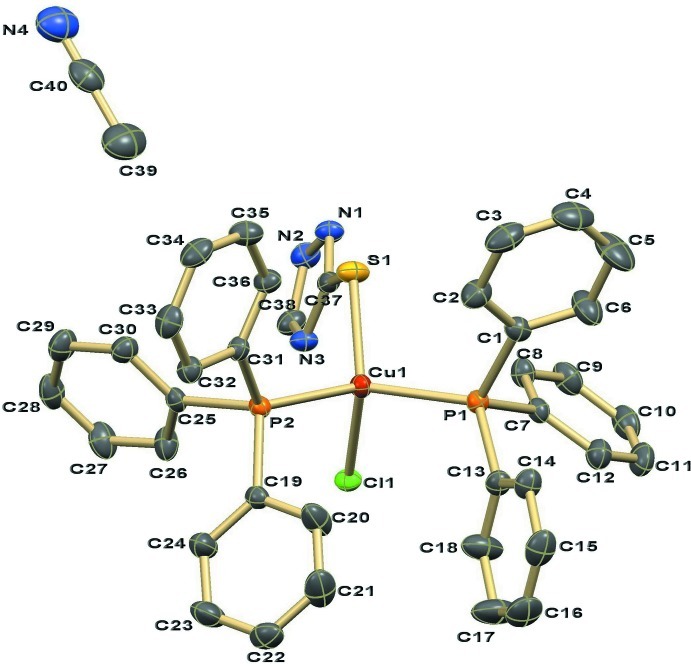
Molecular structure of the title complex with displacement ellipsoids plotted at the 30% probability level.

**Fig. 2.**
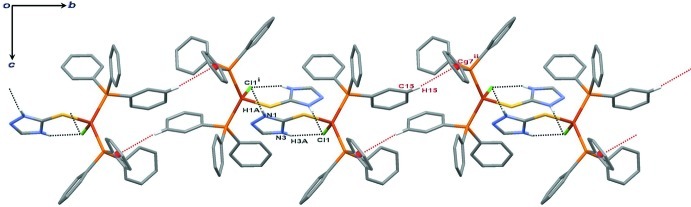
The interactions sheet of the title complex plot down a axis.

**Fig. 3.**
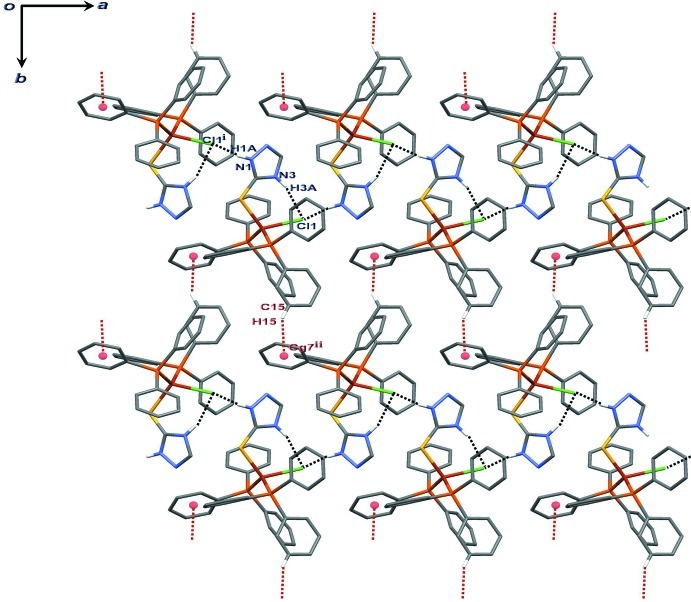
The two-dimensional sheet of hydrogen bond, N–H···Cl, and C–H···π interaction of the title complex plotted parallel to (001). All H atoms not involving the interactions are omitted.

## supplementary crystallographic information

### Comment

The mixed ligand metal(I) complexes of IB group have been studied and characterized due to various properties such as magnetism (Oshio *et al.*, 1996), mocroporus (Zhang & Chen, 2003) and luminescent properties (Vitale & Ford, 2001). Besides, some mixed ligand of copper(I) with drug has been studied (Chen *et al.*, 2001).

For the 1,2,4-triazole-2-thione and its derivatives group have been used as an active ligand to coordinate metals with interesting intrinsic properties (Sen *et al.*, 1996; Zhang *et al.*, 2008). This study reports the crystal structure analysis and self-assembly of the tiltle complex base on mixed ligand copper(I) complex containing triphenylphosphosphane (PPh_3_) and 1*H*-1,2,4-triazole-2-thione (C_2_H_3_N_3_S).

The title compound, [Cu(C_2_H_3_N_3_S)(PPh_3_)_2_Cl].CH_3_CN, is a mononuclear complex. The asymetric unit of the complex contains one formula unit with no crystallographically imposed symmetry and a non-coordinating acetonitrile solvent molecule (Fig.1), in which Cu center is in distorted tetrahedral geometry coordinated by two P atoms of two PPh_3_ molecules, one S atom from C_2_H_3_N_3_S molecule and one Cl atom. Similar to those copper(I) complexes coordinating with mixed PPh_3_/ heterocyclic thione and Cl ligands, the geometry around copper center and the coordination modes are in agreement with the previous reports (Aslanidis *et al.*, 1998; Li *et al.*, 2004; Lobana *et al.*,2008).

The short non-bonding distance between N at 3-positon of triazole ring and Cl atom (N3–H3A···Cl1) in the molecule can be accepted as an intra-molecular hydrogen bond with the N3···Cl1 distance = 3.183 (3) Å and the N3–H3A···Cl1 bond angle = 155 (5)°. In crystal packing, the one-dimensional interaction chain along [100] is connected by inter- hydrogen bonding interactions, N–H···Cl, between N at 1-position of triazole ring and the Cl atom of neighbouring molecule (N1···Cl1^i^ = 3.154 (3) Å; i: *x* + 1/2, -*y* + 3/2, -*z* + 2). In addition, each chain is further linked to each other to form two-dimensional network parallel to (001) due to C—H···π interactions between C15 of phenyl ring and the *Cg*7 centroid (*Cg*7: C31–C32–C33–C34–C35–C36) of the nearby phenyl ring of adjacent molecule with the C···*Cg*7^ii^ distance of 3.749 (4) Å (ii: -*x* - 1, *y* + 5/2, -*z* + 5/2). Two perspective views of intra- and inter-interactions are depicted in Fig. 2 and 3.

### Experimental

A mixture of CuCl (0.15 g: 1.50 mmol), C_2_H_3_N_3_S (0.15 g: 1.48 mmol) and PPh_3_ (0.80: 3.05 mmol) in acetronitrile 30 ml was refluxed for 4 h. Then, the fitrate was kept to evaporate at room temperature over night. The polygon colorless crystals were obtained. The complex melts at 140–142°C.

### Refinement

All carbon H-atoms of triazole ring and phenyl ring were placed in calculated positions (C—H = 0.93 Å) and were included in the refinement in the riding-model approximation, with *U*_iso_(H) = 1.2*U*_eq_(C). The hydrogen atoms of N atoms are located in the difference map and restrained, N—H = 0.86 Å with *U*_iso_(H) = 1.2*U*_eq_(N).

### Crystal data

**Table:**

[CuCl(C_2_H_3_N_3_S)(C_18_H_15_P)_2_]·C_2_H_3_N	*F*(000) = 1584
*M**_r_* = 765.72	*D*_x_ = 1.355 Mg m^−^^3^
Orthorhombic, *P*2_1_2_1_2_1_	Mo *K*α radiation, λ = 0.71073 Å
Hall symbol: P 2ac 2ab	Cell parameters from 5583 reflections
*a* = 10.2348 (4) Å	θ = 2.2–21.3°
*b* = 16.4046 (7) Å	µ = 0.83 mm^−^^1^
*c* = 22.3632 (9) Å	*T* = 293 K
*V* = 3754.7 (3) Å^3^	Polyhedron, colourless
*Z* = 4	0.27 × 0.18 × 0.09 mm

### Data collection

**Table:**

Bruker APEX CCD diffractometer	6602 independent reflections
Radiation source: fine-focus sealed tube	6040 reflections with *I* > 2σ(*I*)
Graphite monochromator	*R*_int_ = 0.044
Frames, each covering 0.3 ° in ω scans	θ_max_ = 25.0°, θ_min_ = 1.5°
Absorption correction: multi-scan (*SADABS*; Bruker, 2003)	*h* = −12→12
*T*_min_ = 0.840, *T*_max_ = 0.928	*k* = −19→19
30968 measured reflections	*l* = −26→26

### Refinement

**Table:**

Refinement on *F*^2^	Secondary atom site location: difference Fourier map
Least-squares matrix: full	Hydrogen site location: inferred from neighbouring sites
*R*[*F*^2^ > 2σ(*F*^2^)] = 0.038	H atoms treated by a mixture of independent and constrained refinement
*wR*(*F*^2^) = 0.086	*w* = 1/[σ^2^(*F*_o_^2^) + (0.0419*P*)^2^ + 0.5093*P*] where *P* = (*F*_o_^2^ + 2*F*_c_^2^)/3
*S* = 1.11	(Δ/σ)_max_ = 0.001
6602 reflections	Δρ_max_ = 0.52 e Å^−^^3^
449 parameters	Δρ_min_ = −0.17 e Å^−^^3^
2 restraints	Absolute structure: Flack (1983), 2890 Friedel pairs
Primary atom site location: structure-invariant direct methods	Flack parameter: −0.001 (11)

### Special details

**Table:**

Geometry. All e.s.d.'s (except the e.s.d. in the dihedral angle between two l.s. planes) are estimated using the full covariance matrix. The cell e.s.d.'s are taken into account individually in the estimation of e.s.d.'s in distances, angles and torsion angles; correlations between e.s.d.'s in cell parameters are only used when they are defined by crystal symmetry. An approximate (isotropic) treatment of cell e.s.d.'s is used for estimating e.s.d.'s involving l.s. planes.
Refinement. Refinement of *F*^2^ against all reflections. The weighted *R*-factor *wR* and goodness of fit *S* are based on *F*^2^, conventional *R*-factors *R* are based on *F*, with *F* set to zero for negative *F*^2^. The threshold expression of *F*^2^ > 2*σ*(*F*^2^) is used only for calculating *R*-factors(gt) *etc*. and is not relevant to the choice of reflections for refinement. *R*-factors based on *F*^2^ are statistically about twice as large as those based on *F*, and *R*- factors based on all data will be even larger.

### Fractional atomic coordinates and isotropic or equivalent isotropic displacement parameters (Å^2^)

**Table:**

	*x*	*y*	*z*	*U*_iso_*/*U*_eq_	
Cu1	0.34524 (4)	0.93751 (2)	0.962685 (16)	0.03712 (11)	
Cl1	0.12918 (7)	0.90194 (5)	0.92963 (4)	0.04224 (19)	
S1	0.46576 (9)	0.82003 (6)	0.98819 (5)	0.0529 (2)	
P1	0.29805 (8)	1.00323 (5)	1.04989 (3)	0.03605 (19)	
P2	0.42531 (8)	0.99058 (5)	0.87575 (4)	0.03542 (19)	
N1	0.4062 (3)	0.65928 (17)	0.98402 (14)	0.0466 (7)	
N2	0.3204 (3)	0.60429 (17)	0.96044 (14)	0.0533 (7)	
N3	0.2723 (3)	0.72977 (17)	0.93421 (14)	0.0440 (7)	
C1	0.4369 (3)	1.0231 (2)	1.09970 (16)	0.0449 (8)	
C2	0.5549 (4)	1.0419 (2)	1.0752 (2)	0.0615 (11)	
H2	0.5638	1.0455	1.0339	0.074*	
C3	0.6623 (4)	1.0557 (3)	1.1121 (3)	0.0848 (15)	
H3	0.7421	1.0703	1.0953	0.102*	
C4	0.6519 (6)	1.0481 (3)	1.1714 (3)	0.0953 (18)	
H4	0.7251	1.0549	1.1955	0.114*	
C5	0.5345 (7)	1.0306 (4)	1.1963 (3)	0.114 (2)	
H5	0.5264	1.0271	1.2377	0.136*	
C6	0.4271 (5)	1.0178 (3)	1.16028 (19)	0.0840 (15)	
H6	0.3469	1.0055	1.1776	0.101*	
C7	0.1835 (3)	0.9471 (2)	1.09762 (14)	0.0401 (8)	
C8	0.1875 (4)	0.8625 (2)	1.09702 (16)	0.0509 (9)	
H8	0.2479	0.8356	1.0730	0.061*	
C9	0.1017 (4)	0.8182 (3)	1.13215 (18)	0.0651 (12)	
H9	0.1063	0.7616	1.1325	0.078*	
C10	0.0106 (4)	0.8570 (3)	1.16626 (18)	0.0691 (12)	
H10	−0.0484	0.8268	1.1888	0.083*	
C11	0.0061 (4)	0.9391 (4)	1.16727 (18)	0.0723 (12)	
H11	−0.0553	0.9651	1.1913	0.087*	
C12	0.0909 (4)	0.9851 (3)	1.13328 (16)	0.0579 (10)	
H12	0.0860	1.0417	1.1343	0.070*	
C13	0.2164 (3)	1.10211 (19)	1.04311 (16)	0.0452 (8)	
C14	0.2650 (4)	1.1740 (2)	1.06457 (17)	0.0543 (10)	
H14	0.3442	1.1741	1.0850	0.065*	
C15	0.1981 (5)	1.2473 (2)	1.0564 (2)	0.0730 (14)	
H15	0.2329	1.2960	1.0706	0.088*	
C16	0.0804 (6)	1.2465 (3)	1.0272 (2)	0.0865 (16)	
H16	0.0348	1.2950	1.0216	0.104*	
C17	0.0295 (6)	1.1749 (3)	1.0060 (3)	0.110 (2)	
H17	−0.0508	1.1744	0.9865	0.132*	
C18	0.0985 (5)	1.1035 (3)	1.0139 (2)	0.0856 (16)	
H18	0.0642	1.0550	0.9990	0.103*	
C19	0.3278 (3)	1.07665 (19)	0.84849 (13)	0.0404 (8)	
C20	0.3033 (5)	1.1388 (2)	0.88831 (19)	0.0784 (15)	
H20	0.3386	1.1364	0.9266	0.094*	
C21	0.2265 (6)	1.2051 (3)	0.8721 (2)	0.0907 (17)	
H21	0.2141	1.2477	0.8989	0.109*	
C22	0.1697 (5)	1.2080 (3)	0.8176 (2)	0.0698 (12)	
H22	0.1157	1.2513	0.8071	0.084*	
C23	0.1932 (5)	1.1460 (3)	0.7780 (2)	0.0815 (14)	
H23	0.1552	1.1474	0.7403	0.098*	
C24	0.2720 (4)	1.0819 (3)	0.79331 (18)	0.0649 (11)	
H24	0.2877	1.0410	0.7654	0.078*	
C25	0.4252 (3)	0.91805 (18)	0.81294 (14)	0.0382 (7)	
C26	0.3092 (4)	0.8834 (3)	0.79486 (16)	0.0606 (11)	
H26	0.2322	0.8961	0.8149	0.073*	
C27	0.3067 (5)	0.8294 (3)	0.74663 (18)	0.0715 (13)	
H27	0.2274	0.8086	0.7333	0.086*	
C28	0.4188 (5)	0.8070 (2)	0.71913 (18)	0.0670 (12)	
H28	0.4168	0.7706	0.6873	0.080*	
C29	0.5353 (5)	0.8384 (2)	0.73845 (19)	0.0668 (12)	
H29	0.6127	0.8224	0.7202	0.080*	
C30	0.5385 (4)	0.8940 (2)	0.78519 (17)	0.0531 (9)	
H30	0.6181	0.9152	0.7978	0.064*	
C31	0.5944 (3)	1.0287 (2)	0.87255 (14)	0.0384 (7)	
C32	0.6363 (4)	1.0808 (2)	0.82716 (16)	0.0479 (8)	
H32	0.5764	1.1023	0.8002	0.057*	
C33	0.7671 (4)	1.1001 (2)	0.82252 (19)	0.0617 (11)	
H33	0.7956	1.1348	0.7923	0.074*	
C34	0.8556 (4)	1.0682 (3)	0.8627 (2)	0.0643 (11)	
H34	0.9441	1.0799	0.8588	0.077*	
C35	0.8137 (4)	1.0195 (2)	0.9080 (2)	0.0603 (10)	
H35	0.8735	0.9990	0.9355	0.072*	
C36	0.6832 (3)	1.0004 (2)	0.91342 (16)	0.0472 (8)	
H36	0.6552	0.9680	0.9450	0.057*	
C37	0.3795 (3)	0.73556 (19)	0.96862 (15)	0.0396 (7)	
C38	0.2396 (4)	0.6494 (2)	0.93112 (17)	0.0514 (9)	
H38	0.1677	0.6295	0.9104	0.062*	
N4	1.0664 (6)	0.7141 (3)	0.8189 (3)	0.1146 (18)	
C39	0.8865 (6)	0.8191 (4)	0.8394 (3)	0.122 (2)	
H39A	0.8616	0.8164	0.8808	0.183*	
H39B	0.8121	0.8074	0.8148	0.183*	
H39C	0.9183	0.8728	0.8305	0.183*	
C40	0.9877 (6)	0.7604 (4)	0.8278 (2)	0.0798 (15)	
H3A	0.219 (4)	0.767 (2)	0.926 (2)	0.096*	
H1A	0.477 (3)	0.646 (3)	1.001 (2)	0.096*	

### Atomic displacement parameters (Å^2^)

**Table:**

	*U*^11^	*U*^22^	*U*^33^	*U*^12^	*U*^13^	*U*^23^
Cu1	0.0398 (2)	0.0367 (2)	0.03486 (19)	−0.00132 (17)	0.00169 (18)	0.00165 (17)
Cl1	0.0320 (4)	0.0448 (4)	0.0499 (5)	−0.0026 (3)	−0.0016 (4)	0.0037 (4)
S1	0.0455 (5)	0.0383 (5)	0.0747 (6)	0.0050 (4)	−0.0170 (5)	−0.0032 (5)
P1	0.0398 (4)	0.0322 (4)	0.0362 (4)	−0.0018 (3)	−0.0010 (3)	−0.0013 (3)
P2	0.0344 (4)	0.0397 (5)	0.0322 (4)	−0.0029 (4)	−0.0011 (3)	0.0033 (4)
N1	0.0458 (17)	0.0364 (16)	0.0577 (19)	0.0051 (14)	−0.0041 (14)	0.0052 (14)
N2	0.0525 (17)	0.0423 (16)	0.0651 (19)	0.0000 (15)	0.0029 (17)	0.0033 (16)
N3	0.0385 (16)	0.0387 (16)	0.0549 (18)	0.0053 (13)	−0.0063 (14)	0.0018 (14)
C1	0.047 (2)	0.0366 (18)	0.051 (2)	−0.0024 (16)	−0.0093 (17)	−0.0040 (15)
C2	0.048 (2)	0.059 (3)	0.077 (3)	−0.0066 (18)	−0.001 (2)	−0.018 (2)
C3	0.046 (2)	0.075 (3)	0.134 (5)	0.000 (2)	−0.005 (3)	−0.029 (3)
C4	0.080 (4)	0.087 (4)	0.119 (5)	0.008 (3)	−0.055 (4)	−0.029 (3)
C5	0.104 (5)	0.156 (6)	0.081 (4)	−0.033 (4)	−0.051 (4)	0.010 (4)
C6	0.071 (3)	0.125 (4)	0.056 (3)	−0.022 (3)	−0.015 (2)	0.004 (3)
C7	0.0415 (19)	0.0448 (19)	0.0340 (16)	−0.0072 (16)	−0.0025 (14)	0.0009 (15)
C8	0.061 (2)	0.047 (2)	0.0440 (19)	−0.0071 (19)	0.0028 (18)	−0.0026 (17)
C9	0.082 (3)	0.059 (2)	0.055 (2)	−0.019 (2)	−0.009 (2)	0.011 (2)
C10	0.065 (3)	0.091 (4)	0.051 (2)	−0.025 (3)	0.004 (2)	0.015 (2)
C11	0.063 (3)	0.102 (4)	0.053 (2)	−0.002 (3)	0.019 (2)	0.001 (3)
C12	0.057 (2)	0.065 (2)	0.052 (2)	−0.005 (2)	0.0072 (19)	−0.003 (2)
C13	0.054 (2)	0.0348 (17)	0.0466 (19)	0.0038 (16)	0.0024 (18)	0.0010 (16)
C14	0.065 (2)	0.039 (2)	0.059 (2)	−0.0034 (18)	0.007 (2)	−0.0043 (17)
C15	0.100 (4)	0.037 (2)	0.082 (3)	0.000 (2)	0.021 (3)	−0.006 (2)
C16	0.104 (4)	0.051 (3)	0.105 (4)	0.033 (3)	0.007 (4)	0.000 (3)
C17	0.093 (4)	0.082 (4)	0.157 (6)	0.039 (3)	−0.049 (4)	−0.013 (4)
C18	0.087 (3)	0.056 (3)	0.114 (4)	0.017 (2)	−0.049 (3)	−0.016 (3)
C19	0.0381 (18)	0.0442 (19)	0.0390 (17)	−0.0013 (15)	0.0002 (15)	0.0077 (14)
C20	0.130 (5)	0.049 (2)	0.056 (2)	0.018 (3)	−0.023 (3)	−0.005 (2)
C21	0.153 (5)	0.046 (2)	0.073 (3)	0.027 (3)	0.009 (3)	−0.001 (2)
C22	0.070 (3)	0.059 (3)	0.080 (3)	0.017 (2)	0.009 (3)	0.027 (2)
C23	0.085 (3)	0.086 (3)	0.073 (3)	0.019 (3)	−0.030 (3)	0.012 (3)
C24	0.076 (3)	0.064 (3)	0.055 (2)	0.020 (2)	−0.014 (2)	−0.0043 (19)
C25	0.0477 (19)	0.0370 (18)	0.0298 (16)	−0.0022 (15)	−0.0008 (15)	0.0044 (13)
C26	0.063 (3)	0.073 (3)	0.046 (2)	−0.017 (2)	0.0061 (19)	−0.013 (2)
C27	0.083 (3)	0.075 (3)	0.056 (3)	−0.026 (3)	−0.008 (2)	−0.014 (2)
C28	0.108 (4)	0.045 (2)	0.048 (2)	−0.002 (2)	0.000 (2)	−0.0103 (18)
C29	0.081 (3)	0.051 (2)	0.069 (3)	0.009 (2)	0.009 (2)	−0.015 (2)
C30	0.055 (2)	0.046 (2)	0.058 (2)	0.0056 (18)	0.0003 (19)	−0.0058 (18)
C31	0.0368 (18)	0.0416 (18)	0.0369 (17)	−0.0023 (14)	0.0049 (15)	−0.0008 (14)
C32	0.046 (2)	0.049 (2)	0.0481 (19)	−0.0044 (17)	0.0041 (17)	0.0025 (16)
C33	0.062 (3)	0.057 (2)	0.065 (2)	−0.016 (2)	0.019 (2)	0.001 (2)
C34	0.040 (2)	0.061 (2)	0.092 (3)	−0.007 (2)	0.012 (2)	−0.009 (2)
C35	0.047 (2)	0.053 (2)	0.081 (3)	0.0003 (19)	−0.012 (2)	0.003 (2)
C36	0.0380 (19)	0.049 (2)	0.055 (2)	−0.0002 (16)	0.0014 (16)	0.0043 (17)
C37	0.0353 (17)	0.0430 (18)	0.0405 (18)	0.0076 (14)	0.0042 (15)	0.0044 (15)
C38	0.042 (2)	0.047 (2)	0.065 (2)	−0.0008 (17)	0.0008 (19)	−0.0066 (18)
N4	0.108 (4)	0.109 (4)	0.127 (4)	0.007 (3)	−0.037 (4)	−0.021 (4)
C39	0.098 (5)	0.127 (5)	0.141 (5)	0.003 (4)	−0.040 (4)	−0.037 (5)
C40	0.080 (4)	0.081 (4)	0.078 (3)	−0.023 (3)	−0.029 (3)	−0.003 (3)

### Geometric parameters (Å, º)

**Table:**

Cu1—P1	2.2802 (9)	C15—H15	0.9300
Cu1—P2	2.2824 (9)	C16—C17	1.370 (7)
Cu1—S1	2.3582 (9)	C16—H16	0.9300
Cu1—Cl1	2.4035 (9)	C17—C18	1.378 (6)
S1—C37	1.700 (3)	C17—H17	0.9300
P1—C13	1.831 (3)	C18—H18	0.9300
P1—C7	1.834 (3)	C19—C24	1.363 (5)
P1—C1	1.834 (4)	C19—C20	1.377 (5)
P2—C19	1.833 (3)	C20—C21	1.390 (6)
P2—C25	1.841 (3)	C20—H20	0.9300
P2—C31	1.841 (3)	C21—C22	1.352 (7)
N1—C37	1.326 (4)	C21—H21	0.9300
N1—N2	1.364 (4)	C22—C23	1.369 (6)
N1—H1A	0.844 (19)	C22—H22	0.9300
N2—C38	1.289 (5)	C23—C24	1.368 (6)
N3—C37	1.343 (4)	C23—H23	0.9300
N3—C38	1.361 (4)	C24—H24	0.9300
N3—H3A	0.835 (19)	C25—C30	1.372 (5)
C1—C6	1.361 (6)	C25—C26	1.377 (5)
C1—C2	1.362 (5)	C26—C27	1.397 (5)
C2—C3	1.393 (6)	C26—H26	0.9300
C2—H2	0.9300	C27—C28	1.352 (6)
C3—C4	1.336 (7)	C27—H27	0.9300
C3—H3	0.9300	C28—C29	1.369 (6)
C4—C5	1.355 (8)	C28—H28	0.9300
C4—H4	0.9300	C29—C30	1.388 (5)
C5—C6	1.379 (7)	C29—H29	0.9300
C5—H5	0.9300	C30—H30	0.9300
C6—H6	0.9300	C31—C36	1.370 (5)
C7—C12	1.386 (5)	C31—C32	1.394 (5)
C7—C8	1.388 (5)	C32—C33	1.380 (5)
C8—C9	1.384 (5)	C32—H32	0.9300
C8—H8	0.9300	C33—C34	1.379 (6)
C9—C10	1.362 (6)	C33—H33	0.9300
C9—H9	0.9300	C34—C35	1.360 (6)
C10—C11	1.347 (7)	C34—H34	0.9300
C10—H10	0.9300	C35—C36	1.378 (5)
C11—C12	1.379 (6)	C35—H35	0.9300
C11—H11	0.9300	C36—H36	0.9300
C12—H12	0.9300	C38—H38	0.9300
C13—C14	1.368 (5)	N4—C40	1.125 (7)
C13—C18	1.373 (6)	C39—C40	1.438 (8)
C14—C15	1.395 (6)	C39—H39A	0.9600
C14—H14	0.9300	C39—H39B	0.9600
C15—C16	1.371 (7)	C39—H39C	0.9600
			
P1—Cu1—P2	128.63 (3)	C16—C17—C18	119.3 (5)
P1—Cu1—S1	106.88 (4)	C16—C17—H17	120.3
P2—Cu1—S1	109.27 (4)	C18—C17—H17	120.3
P1—Cu1—Cl1	100.54 (3)	C13—C18—C17	121.6 (5)
P2—Cu1—Cl1	99.26 (3)	C13—C18—H18	119.2
S1—Cu1—Cl1	110.93 (3)	C17—C18—H18	119.2
C37—S1—Cu1	109.39 (11)	C24—C19—C20	117.5 (3)
C13—P1—C7	101.60 (16)	C24—C19—P2	125.3 (3)
C13—P1—C1	104.29 (16)	C20—C19—P2	117.0 (3)
C7—P1—C1	103.35 (15)	C19—C20—C21	120.9 (4)
C13—P1—Cu1	116.40 (12)	C19—C20—H20	119.5
C7—P1—Cu1	113.32 (11)	C21—C20—H20	119.5
C1—P1—Cu1	116.05 (12)	C22—C21—C20	120.4 (4)
C19—P2—C25	104.12 (14)	C22—C21—H21	119.8
C19—P2—C31	103.71 (15)	C20—C21—H21	119.8
C25—P2—C31	100.96 (15)	C21—C22—C23	118.7 (4)
C19—P2—Cu1	112.43 (10)	C21—C22—H22	120.6
C25—P2—Cu1	113.78 (10)	C23—C22—H22	120.6
C31—P2—Cu1	120.00 (11)	C24—C23—C22	120.9 (4)
C37—N1—N2	113.0 (3)	C24—C23—H23	119.6
C37—N1—H1A	122 (4)	C22—C23—H23	119.6
N2—N1—H1A	124 (4)	C19—C24—C23	121.5 (4)
C38—N2—N1	103.2 (3)	C19—C24—H24	119.3
C37—N3—C38	107.3 (3)	C23—C24—H24	119.3
C37—N3—H3A	128 (4)	C30—C25—C26	118.5 (3)
C38—N3—H3A	122 (4)	C30—C25—P2	122.0 (3)
C6—C1—C2	118.7 (4)	C26—C25—P2	119.4 (3)
C6—C1—P1	122.4 (3)	C25—C26—C27	120.3 (4)
C2—C1—P1	118.9 (3)	C25—C26—H26	119.9
C1—C2—C3	119.9 (4)	C27—C26—H26	119.9
C1—C2—H2	120.1	C28—C27—C26	120.5 (4)
C3—C2—H2	120.1	C28—C27—H27	119.7
C4—C3—C2	120.6 (5)	C26—C27—H27	119.7
C4—C3—H3	119.7	C27—C28—C29	119.6 (4)
C2—C3—H3	119.7	C27—C28—H28	120.2
C3—C4—C5	120.0 (5)	C29—C28—H28	120.2
C3—C4—H4	120.0	C28—C29—C30	120.4 (4)
C5—C4—H4	120.0	C28—C29—H29	119.8
C4—C5—C6	119.9 (5)	C30—C29—H29	119.8
C4—C5—H5	120.1	C25—C30—C29	120.6 (4)
C6—C5—H5	120.1	C25—C30—H30	119.7
C1—C6—C5	120.9 (5)	C29—C30—H30	119.7
C1—C6—H6	119.5	C36—C31—C32	119.3 (3)
C5—C6—H6	119.5	C36—C31—P2	118.8 (3)
C12—C7—C8	118.4 (3)	C32—C31—P2	121.7 (3)
C12—C7—P1	123.1 (3)	C33—C32—C31	119.6 (4)
C8—C7—P1	118.5 (3)	C33—C32—H32	120.2
C9—C8—C7	120.0 (4)	C31—C32—H32	120.2
C9—C8—H8	120.0	C34—C33—C32	120.0 (4)
C7—C8—H8	120.0	C34—C33—H33	120.0
C10—C9—C8	120.5 (4)	C32—C33—H33	120.0
C10—C9—H9	119.8	C35—C34—C33	120.2 (4)
C8—C9—H9	119.8	C35—C34—H34	119.9
C11—C10—C9	120.0 (4)	C33—C34—H34	119.9
C11—C10—H10	120.0	C34—C35—C36	120.3 (4)
C9—C10—H10	120.0	C34—C35—H35	119.9
C10—C11—C12	121.1 (4)	C36—C35—H35	119.9
C10—C11—H11	119.5	C31—C36—C35	120.5 (4)
C12—C11—H11	119.5	C31—C36—H36	119.7
C11—C12—C7	120.1 (4)	C35—C36—H36	119.7
C11—C12—H12	119.9	N1—C37—N3	104.5 (3)
C7—C12—H12	119.9	N1—C37—S1	126.5 (3)
C14—C13—C18	118.2 (3)	N3—C37—S1	129.0 (3)
C14—C13—P1	124.7 (3)	N2—C38—N3	111.9 (3)
C18—C13—P1	117.1 (3)	N2—C38—H38	124.1
C13—C14—C15	121.3 (4)	N3—C38—H38	124.1
C13—C14—H14	119.4	C40—C39—H39A	109.5
C15—C14—H14	119.4	C40—C39—H39B	109.5
C16—C15—C14	119.0 (4)	H39A—C39—H39B	109.5
C16—C15—H15	120.5	C40—C39—H39C	109.5
C14—C15—H15	120.5	H39A—C39—H39C	109.5
C17—C16—C15	120.5 (4)	H39B—C39—H39C	109.5
C17—C16—H16	119.7	N4—C40—C39	179.7 (8)
C15—C16—H16	119.7		
			
P1—Cu1—S1—C37	116.24 (13)	P1—C13—C14—C15	178.5 (3)
P2—Cu1—S1—C37	−100.90 (13)	C13—C14—C15—C16	1.1 (7)
Cl1—Cu1—S1—C37	7.53 (13)	C14—C15—C16—C17	−0.3 (8)
P2—Cu1—P1—C13	41.73 (14)	C15—C16—C17—C18	−0.6 (10)
S1—Cu1—P1—C13	174.88 (13)	C14—C13—C18—C17	−0.1 (7)
Cl1—Cu1—P1—C13	−69.26 (13)	P1—C13—C18—C17	−179.5 (5)
P2—Cu1—P1—C7	159.03 (12)	C16—C17—C18—C13	0.8 (10)
S1—Cu1—P1—C7	−67.83 (12)	C25—P2—C19—C24	−0.6 (4)
Cl1—Cu1—P1—C7	48.03 (12)	C31—P2—C19—C24	104.7 (3)
P2—Cu1—P1—C1	−81.59 (13)	Cu1—P2—C19—C24	−124.2 (3)
S1—Cu1—P1—C1	51.56 (13)	C25—P2—C19—C20	175.6 (3)
Cl1—Cu1—P1—C1	167.42 (13)	C31—P2—C19—C20	−79.2 (4)
P1—Cu1—P2—C19	−53.12 (13)	Cu1—P2—C19—C20	51.9 (4)
S1—Cu1—P2—C19	174.57 (12)	C24—C19—C20—C21	−1.3 (7)
Cl1—Cu1—P2—C19	58.44 (12)	P2—C19—C20—C21	−177.8 (4)
P1—Cu1—P2—C25	−171.18 (12)	C19—C20—C21—C22	2.9 (8)
S1—Cu1—P2—C25	56.52 (12)	C20—C21—C22—C23	−2.4 (8)
Cl1—Cu1—P2—C25	−59.61 (12)	C21—C22—C23—C24	0.4 (8)
P1—Cu1—P2—C31	69.18 (13)	C20—C19—C24—C23	−0.7 (7)
S1—Cu1—P2—C31	−63.12 (13)	P2—C19—C24—C23	175.5 (4)
Cl1—Cu1—P2—C31	−179.25 (13)	C22—C23—C24—C19	1.2 (8)
C37—N1—N2—C38	−1.1 (4)	C19—P2—C25—C30	119.2 (3)
C13—P1—C1—C6	88.1 (4)	C31—P2—C25—C30	11.9 (3)
C7—P1—C1—C6	−17.8 (4)	Cu1—P2—C25—C30	−118.1 (3)
Cu1—P1—C1—C6	−142.5 (4)	C19—P2—C25—C26	−64.1 (3)
C13—P1—C1—C2	−93.5 (3)	C31—P2—C25—C26	−171.4 (3)
C7—P1—C1—C2	160.6 (3)	Cu1—P2—C25—C26	58.6 (3)
Cu1—P1—C1—C2	36.0 (3)	C30—C25—C26—C27	−4.3 (6)
C6—C1—C2—C3	−0.3 (6)	P2—C25—C26—C27	178.9 (3)
P1—C1—C2—C3	−178.8 (3)	C25—C26—C27—C28	3.5 (7)
C1—C2—C3—C4	2.1 (7)	C26—C27—C28—C29	−0.7 (7)
C2—C3—C4—C5	−3.1 (8)	C27—C28—C29—C30	−1.2 (7)
C3—C4—C5—C6	2.2 (10)	C26—C25—C30—C29	2.4 (5)
C2—C1—C6—C5	−0.5 (8)	P2—C25—C30—C29	179.2 (3)
P1—C1—C6—C5	177.9 (5)	C28—C29—C30—C25	0.4 (6)
C4—C5—C6—C1	−0.4 (10)	C19—P2—C31—C36	149.0 (3)
C13—P1—C7—C12	−19.7 (3)	C25—P2—C31—C36	−103.3 (3)
C1—P1—C7—C12	88.2 (3)	Cu1—P2—C31—C36	22.6 (3)
Cu1—P1—C7—C12	−145.4 (3)	C19—P2—C31—C32	−35.7 (3)
C13—P1—C7—C8	158.7 (3)	C25—P2—C31—C32	71.9 (3)
C1—P1—C7—C8	−93.3 (3)	Cu1—P2—C31—C32	−162.2 (2)
Cu1—P1—C7—C8	33.1 (3)	C36—C31—C32—C33	2.8 (5)
C12—C7—C8—C9	−0.9 (5)	P2—C31—C32—C33	−172.4 (3)
P1—C7—C8—C9	−179.5 (3)	C31—C32—C33—C34	−0.1 (6)
C7—C8—C9—C10	1.8 (6)	C32—C33—C34—C35	−2.0 (6)
C8—C9—C10—C11	−1.9 (7)	C33—C34—C35—C36	1.4 (6)
C9—C10—C11—C12	1.3 (7)	C32—C31—C36—C35	−3.4 (5)
C10—C11—C12—C7	−0.5 (6)	P2—C31—C36—C35	171.9 (3)
C8—C7—C12—C11	0.3 (5)	C34—C35—C36—C31	1.3 (6)
P1—C7—C12—C11	178.8 (3)	N2—N1—C37—N3	0.4 (4)
C7—P1—C13—C14	115.5 (3)	N2—N1—C37—S1	−178.8 (2)
C1—P1—C13—C14	8.3 (4)	C38—N3—C37—N1	0.4 (4)
Cu1—P1—C13—C14	−120.9 (3)	C38—N3—C37—S1	179.5 (3)
C7—P1—C13—C18	−65.0 (4)	Cu1—S1—C37—N1	−168.9 (3)
C1—P1—C13—C18	−172.2 (3)	Cu1—S1—C37—N3	12.1 (3)
Cu1—P1—C13—C18	58.6 (4)	N1—N2—C38—N3	1.3 (4)
C18—C13—C14—C15	−0.9 (6)	C37—N3—C38—N2	−1.1 (4)

### Hydrogen-bond geometry (Å, º)

Cg7 is the centroid of the C31–C36 ring.

**Table:**

*D*—H···*A*	*D*—H	H···*A*	*D*···*A*	*D*—H···*A*
N3—H3*A*···Cl1	0.84 (2)	2.41 (3)	3.183 (3)	155 (5)
N1—H1*A*···Cl1^i^	0.84 (2)	2.34 (2)	3.154 (3)	163 (5)
C15—H15···*Cg*7^ii^	0.93	2.88	3.749 (4)	155

Symmetry codes: (i) *x*+1/2, −*y*+3/2, −*z*+2; (ii) −*x*−1, *y*+5/2, −*z*+5/2.

## References

[bb1] Aslanidis, P., Hadjikakou, S. K., Karagiannidis, P. & Cox, P. J. (1998). *Inorg. Chim. Acta*, **271**, 243–247.

[bb2] Bruker (2003). *SMART*, *SAINT* and *SADABS* Bruker AXS Inc., Madison, Wisconsin, USA.

[bb3] Chen, Z.-F., Li, B.-Q., Xie, Y.-R., Xiong, R.-G., You, X.-Z. & Feng, X.-L. (2001). *Inorg. Chem. Commun.* **4**, 346–349.

[bb4] Flack, H. D. (1983). *Acta Cryst.* A**39**, 876–881.

[bb5] Henary, M., Wootton, J. L., Khan, S. I. & Zink, J. I. (1997). *Inorg. Chem.* **36**, 796–801.

[bb6] La-o, L., Pakawatchai, C., Saithong, S. & Skelton, B. W. (2009). *Acta Cryst.* E**65**, m926. 10.1107/S1600536809026798 PMC2977114 21583381

[bb7] Li, D., Shi, W.-J., Wu, T. & Ng, S. W. (2004). *Acta Cryst.* E**60**, m776–m777.

[bb8] Lobana, T. S., Sultana, R. & Hundal, G. (2008). *Polyhedron*, **27**, 1008–1016.

[bb9] Macrae, C. F., Bruno, I. J., Chisholm, J. A., Edgington, P. R., McCabe, P., Pidcock, E., Rodriguez-Monge, L., Taylor, R., van de Streek, J. & Wood, P. A. (2008). *J. Appl. Cryst.* **41**, 466–470.

[bb10] Oshio, H., Watanabe, T., Ohto, A., Ito, T. & Masuda, H. (1996). *Inorg. Chem.* **35**, 472–479. 10.1021/ic950663v 11666232

[bb11] Sen, A. K., Dubey, S. N. & Squattrito, P. J. (1996). *Acta Cryst.* C**52**, 865–868.

[bb12] Sheldrick, G. M. (2008). *Acta Cryst.* A**64**, 112–122. 10.1107/S0108767307043930 18156677

[bb13] Skoulika, S., Aubry, A., Karagiannidis, P., Aslanidis, P. & Papastefanou, S. (1991). *Inorg. Chim. Acta*, **183**, 207–211.

[bb14] Spek, A. L. (2009). *Acta Cryst.* D**65**, 148–155. 10.1107/S090744490804362X PMC2631630 19171970

[bb15] Vitale, M. & Ford, P. C. (2001). *Coord. Chem. Rev.* **219–221**, 3–16.

[bb16] Westrip, S. P. (2010). *J. Appl. Cryst.* **43**, 920–925.

[bb17] Zhang, X.-M. & Chen, X.-M. (2003). *Eur. J. Inorg. Chem.* pp. 413–417.

[bb18] Zhang, R.-B., Li, Z.-J., Cheng, J.-K., Qin, Y.-Y., Zhang, J. & Yao, Y.-G. (2008). *Cryst. Growth Des.* **8**, 2562–2573.

